# Encapsulation of Methanotrophs within a Polymeric Matrix Containing Copper- and Iron-Based Nanoparticles to Enhance Methanol Production from a Simulated Biogas

**DOI:** 10.3390/polym15183667

**Published:** 2023-09-06

**Authors:** Sanjay K. S. Patel, Rahul K. Gupta, In-Won Kim, Jung-Kul Lee

**Affiliations:** Department of Chemical Engineering, Konkuk University, 120 Neungdong-ro, Gwangjin-gu, Seoul 05029, Republic of Koreaguptarahul9m@gmail.com (R.K.G.)

**Keywords:** biogas, greenhouse gases, encapsulation, methanol, methanotrophs, polymeric matrix

## Abstract

The production of renewable energy or biochemicals is gaining more attention to minimize the emissions of greenhouse gases such as methane (CH_4_) and carbon dioxide for sustainable development. In the present study, the influence of copper (Cu)- and iron (Fe)-based nanoparticles (NPs), such as Cu, Fe_3_O_4_, and CuFe_2_O_4_, was evaluated during the growth of methanotrophs for inoculum preparation and on the development of a polymeric-matrix-based encapsulation system to enhance methanol production from simulated biogas (CH_4_ and CO_2_). The use of simulated biogas feed and the presence of NP-derived inoculums produce a remarkable enhancement in methanol production up to 149% and 167% for *Methyloferula stellata* and *Methylocystis bryophila* free-cells-based bioconversion, respectively, compared with the use of pure CH_4_ as a control feed during the growth stage. Furthermore, these methanotrophs encapsulated within a polymeric matrix and NPs-based systems exhibited high methanol production of up to 156%, with a maximum methanol accumulation of 12.8 mmol/L over free cells. Furthermore, after encapsulation, the methanotrophs improved the stability of residual methanol production and retained up to 62.5-fold higher production potential than free cells under repeated batch reusability of 10 cycles. In the presence of CH_4_ vectors, methanol production by *M. bryophila* improved up to 16.4 mmol/L and retained 20% higher recycling stability for methanol production in paraffin oil. These findings suggest that Cu and Fe NPs can be beneficially employed with a polymeric matrix to encapsulate methanotrophs and improve methanol production.

## 1. Introduction

Methanotrophs are microbes that utilize methane (CH_4_) for growth and produce numerous value-added bioproducts such as methanol, 2,3-butanediol, ectoine, lactic acid, and biopolymers [1,2]. In recent years, the analysis of methanotrophic pathways and their metabolic engineering has significantly expanded to produce bioproducts from CH_4_ and carbon dioxide (CO_2_) as greenhouse gases (GHGs) [3,4]. Methanol production by methanotrophs can be beneficial over CH_4_ as an energy source due to its ~400 times higher density. Methanol can readily be transported or stored under ambient conditions compared with CH_4_. [5]. Methane monooxygenases (MMOs) catalyze CH_4_ to methanol in methanotrophs. Subsequently, during methanotroph metabolism, methanol is converted to formaldehyde by methanol dehydrogenase (MDH), formaldehyde is converted to formate by formaldehyde dehydrogenase and finally to CO_2_ by formate dehydrogenase [5]. The accumulation of methanol is influenced by the type of methanotroph and the conversion physiological conditions, such as the inoculum, incubation period, pH, temperature, and nature of the feed, such as pure CH_4_ or CO_2_, and biogas (CH_4_ and CO_2_) [6,7,8]. The low growth and methanol yield of methanotrophs are mainly associated with the partial solubility of CH_4_ in media and the successive metabolization of accumulated methanol via the partial inactivation of MDH activity during conversion within the cells by MDH inhibitors such as magnesium chloride (MgCl_2_) and phosphate buffer [9,10]. 

Strategies such as metabolic engineering and immobilization of methanotrophs have been shown to improve methanol production [11,12]. However, engineering methanotrophs can be challenging owing to the complicated procedures involved, especially homologous expression [12]. Using immobilized cells has been proven to be more beneficial than using free cells for repeated batch conversion and easy separation from the reaction mixture [6,11]. The immobilization of whole cells on solid supports is primarily limited by the non-eco-friendly chemical modification of the support for the binding of cells, including covalent immobilization, substantial loss of biocatalytic activity after immobilization, and low recycling stability of immobilized cells [6,11,13]. Therefore, the adaptation of suitable immobilization procedures can be beneficial to achieve higher methanol production because of the availability of a broad range of immobilization supports, such as biomass and polymeric or synthetic materials, and techniques, including adsorption, encapsulation, and covalent methods, for the attachment of cells to supports [11,14,15]. In addition, methanotroph growth and MMO activity are highly affected by metals such as copper (Cu) and iron (Fe) [16]. 

Nanoparticles (NPs) significantly affect cellular properties during growth or bioconversion applications [17]. Polymeric materials, such as silica, are desirable for supporting immobilized cells through encapsulation because of their high biocompatibility [18]. Therefore, the use of Cu- and Fe-based NPs and polymeric matrices is desirable for enhancing methanol production. In this study, the influence of Cu- and Fe-based NPs, such as Cu, Fe_3_O_4,_ and CuFe_2_O_4_, was evaluated during the growth of methanotrophs to prepare competent inocula to enhance methanol biotransformation using simulated biogas as the feed. Furthermore, methanotrophs were encapsulated within a polymeric matrix containing NPs to improve cell stability and methanol production. Finally, the CH_4_ vectors allowed greater conversion under repeated recycling in batch-mode production. This is the first study to demonstrate enhanced methanol production from simulated biogas using a combined polymer and NP-based matrix approach for encapsulating precultured methanotrophs in the presence of NPs. 

## 2. Materials and Methods

### 2.1. Materials and Cultures

NPs of Cu (25 nm), CuFe_2_O_4_ (100 nm), Pluronic^®^ P-123 tri-block polymer [poly(ethylene glycol)-block-poly(propylene glycol)-block-poly(ethylene glycol)]), tetraethylorthosilicate (TEOS), polyethylene glycol, tetrazotized-o-dianisidine, 2,6-dichlorophenol-indophenol (DCPIP), phenazine methosulfate, Brij 35, paraffin oil, and silicon oil were procured from Sigma-Aldrich (Burlington, MA, USA). Fe_3_O_4_ (<100 nm) was procured from Nanostructured and Amorphous Materials, Inc. (Houston, TX, USA). All other chemicals used in this study were procured from commercial sources. High-purity gases were purchased from NK Co., Ltd. (Busan, Republic of Korea). Methanotrophs were purchased from the German Collection of Microorganisms and Cell Cultures (Braunschweig, Germany) [19]. 

### 2.2. Influence of NPs on the Growth of Methanotrophs for Methanol Production

Methanotrophs were cultivated in a 1 L flask (Duran-Schott, Germany) with an air-tight screw cap (Suba seal, Sigma-Aldrich, USA) and with 200 mL of nitrate mineral salt (NMS) medium containing CH_4_ (30%, *v*/*v*) or simulated biogas (CH_4_, 30% (*v*/*v*) and CO_2_, 7.5% (*v*/*v*)) as feed for up to 5 days at 30 °C, with shaking, in the presence of various NPs (0.01 mg/mL): Cu, Fe_3_O_4_, or CuFe_2_O_4_ [20]. The cell dry mass was analyzed using constant-weight measurements at 70 °C [19]. The production of methanol in batch cultures was examined in 120 mL bottles (Sigma-Aldrich, USA) containing 20 mL of phosphate buffer (100 mM, pH 6.8) and MgCl_2_ (as MDH inhibitor), using methanotrophs inoculum at 3.0 mg DCM/mL from simulated biogas (CH_4_, 30% (*v*/*v*), and CO_2_, 15% (*v*/*v*)) for incubation at 30 °C for 48 h [21].

### 2.3. Encapsulation of Whole Cells in Polymeric Matrix Containing Nanoparticles for Methanol Production and Biocatalytic Activity

Methanotroph encapsulation was performed using a synthetic precursor solution (20 mL), which was a mixture of TEOS/Pluronic^®^ P-123/H_2_O/ethanol/HCl/glycerol (molar ratio:1:0.015:5.3:18.1:0.3:1.13; pH 5.0) and methanotrophs (cultured in NPs (0.01 mg/mL) containing NMS media) and fresh culture (1 mg of DCM/mL, 40 mL) [18]. Briefly, the fresh inoculum was mixed with the precursor solution, followed by incubation at 30 °C for 2 h under continuous stirring. The encapsulated cells were washed with distilled water and buffer solution to remove any unbound cells. Furthermore, different NP (Cu, Fe_3_O_4_, or CuFe_2_O_4_) concentrations (0.01–0.05 mg/mL) were added to the synthetic precursor solution (TEOS/Pluronic^®^ P-123/H_2_O/ethanol/HCl/glycerol) during cell encapsulation to improve methanol production. The biocatalytic activities of the MMOs and the MDH of the methanotrophs were evaluated via naphthalene oxidation and DCPIP reduction via phenazine-methosulfate, respectively [20]. All encapsulated cells were stored at 4 °C until further use. 

### 2.4. Influence of Process Parameters on Methanol Production by Encapsulated Methanotrophs

The effects of pH (6.0–8.0), followed by temperature (25–40 °C), were evaluated for methanol production using free and encapsulated cells from simulated biogas (CH_4_, 30% (*v*/*v*), and CO_2_, 15% (*v*/*v*)) at 30 °C and 48 h of incubation. 

### 2.5. Reusability Measurements

The repeated-batch-mode methanol production by free and encapsulated *M. bryophila* within a CuFe_2_O_4_-containing polymeric matrix from simulated biogas at 30 °C and 24 h of incubation was measured. Free and encapsulated cells were retrieved via centrifugation and used as the inoculum in the next batch cycle [22]. The initial efficiency of the methanol production or MMO activity of free and polymeric-matrix-encapsulated cells was considered to be 100%.

### 2.6. Influence of CH_4_ Vectors on Methanol Production by Encapsulated M. bryophila

The effects of CH_4_ vectors (5%, *w/v* or *v*/*v*), including Brij 35 repeated-batch-mode methanol production by free or encapsulated *M. bryophila* from simulated biogas at 30 °C and 48 h of incubation were determined. Furthermore, to enhance methanol production, the reusability of up to 10 cycles for free and encapsulated *M. bryophila* within CuFe_2_O_4_-containing polymeric matrix cells in the presence of paraffin oil (5%, *v*/*v*) was evaluated. 

### 2.7. Instrumental Analysis

All absorbance measurements were performed spectrophotometrically using a 6705 UV/Vis spectrophotometer (Jenway Scientific, Staffordshire, UK) [22]. The methanol concentration was assessed using an Agilent 7890A chromatography system equipped with a flame ionization detector and an Agilent 19091 J-413 (HP-5) column (Santa Clara, CA, USA) [19]. Validation of whole-cell encapsulation within CuFe_2_O_4_-containing polymeric matrix was performed via field-emission scanning electron microscopy (FE-SEM, JEOL, Tokyo, Japan) [21]. X-ray diffraction (XRD) analysis was performed using an X’pert PRO MPD, Malvern PANalytical, Malvern, UK). All presented values are based on three experimental replicates. 

## 3. Results and Discussion

### 3.1. Influence of NPs on the Growth of Methanotrophs to Enhance Methanol Production

**Figure 1:**
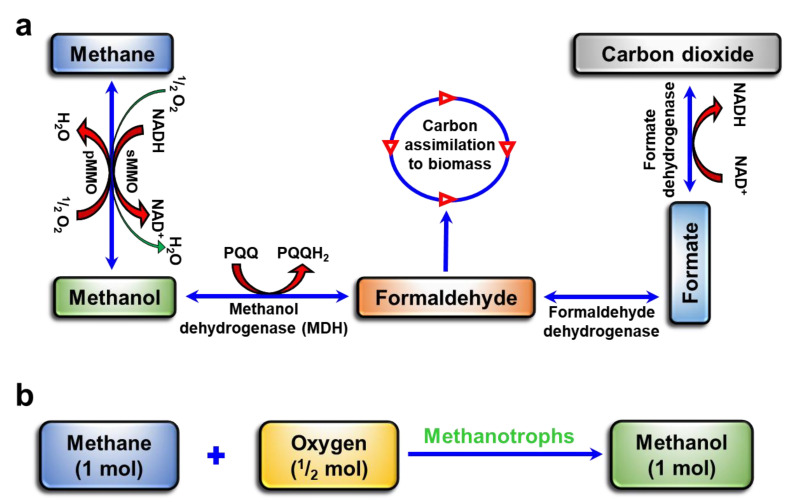
(**a**) Methane metabolism in methanotrophs (sMMO, soluble methane monooxygenase; pMMO, particulate methane monooxygenase; NADH, nicotinamide adenine dinucleotide; and PQQ, pyrroloquinoline quinone), and (**b**) stoichiometry reaction for the conversion of methane to methanol by methanotrophs (0.5 mol of O_2_ is required to produce 1 mol of methanol).

The NPs have been widely used in various renewable energy sources to modulate physiological properties such as the biocatalytic activity of whole cells containing metallic enzymes [17,23]. Pure CH_4_ has been widely used as a feed for methanotrophs to prepare inocula for converting simulated biogas into methanol [5,24]. Therefore, the effect of NPs based on Cu and Fe on the growth of methanotrophs to enhance methanol conversion from biogas was evaluated in this study because these metals are involved in the MMO biocatalytic activity of methanotrophs [16,25]. With CH_4_ (30%) as the feed (control), *M. bryophila* and *M. stellata* had average specific growth rates (µ) of 0.013 and 0.008 h^−^^1^, respectively, for up to 5 days of incubation (Table 1). The addition of CO_2_ (7.5%) at a 4:1 ratio during culture resulted in a slight increase in µ of up to 15% for these methanotrophs. The presence of various NPs (0.01 mg/mL) of Cu, Fe_3_O_4,_ and CuFe_2_O_4_ resulted in higher values of up to 0.021 and 0.015 h^−^^1^ for methanotrophs using simulated biogas versus 0.019 and 0.012 h^−^^1^ using CH_4_ as a feed. In the presence of these NPs, µ improved by as much as 46.1% and 66.7% for *M. bryophila* and *M. stellata*, respectively. The higher µ in the presence of NPs was supported by better substrate utilization of 23.3% and 35.4% compared with the control values of 17.9% and 22.4% for *M. bryophila* and *M. stellata*, respectively (Table S1). The metabolism of CH_4_ and methanol accumulation in methanotrophs are illustrated in Figure 1. The CH_4-_feed-based inoculum as a control resulted in methanol production of 4.90 mmol/L (98.0 µmol) for *M. bryophila* and 3.11 mmol/L (62.2 µmol) for *M. stellata* after 48 h of incubation from simulated biogas as a feed (Table 1). Previously, the maximum methanol concentration accumulations of 0.02 and 0.71 mmol/L were reported from simulated biogas using CH_4_-based inocula of *Methylosinus trichosporium* IMV 3011 and *Methylosinus sporium* KCTC 22312, respectively [9,26]. Up to 11% higher methanol production was observed for these methanotrophs by shifting the feed from pure CH_4_ to simulated biogas. The inocula of *M. bryophila* derived from NP media exhibited a remarkable enhancement in methanol production of up to 48, 25, and 67% for Cu, Fe_3_O_4,_ and CuFe_2_O_4_, respectively. Similarly, *M. stellata* exhibited a relative efficiency of up to 149%. The methanol production profiles showed that the optimum incubation was recorded at 48 h using CH_4_- or simulated-biogas-based inocula (Figure S1). At a longer incubation of 96 h, the decline in methanol accumulation could have be linked to the subsequent metabolism of methanol by methanotrophs owing to partial MDH inhibition. The maximum methanol production enhancement was 8.18 mmol/L(164 µmol) from 4.98 mmol/L for *M. bryophila* and 4.63 mmol/L (92.6 µmol) from 3.11 mmol/L for *M. stellata* using simulated biogas as the feed with CuFe_2_O_4_-based inocula relative to the controls are shown in Figure S1. Previously, the effect of cerium on *Methylacidiphilum fumariolicum* growth during methanol production was reported, with a maximum methanol concentration accumulation of 4.9 mmol/L from simulated biogas [23]. In contrast, no methanol accumulation was recorded for *M. fumariolicum* using pure CH_4_ as the feed in the presence of cerium (1 µM). The results of this study suggest that the Cu- and Fe-based NPs positively influence the growth and methanol production by *M. bryophila* and *M. stellata* using pure CH_4_ or simulated biogas as the feed. 

### 3.2. Encapsulation of Methanotrophs within Polymeric Matrix Containing NPs

**Figure 2:**
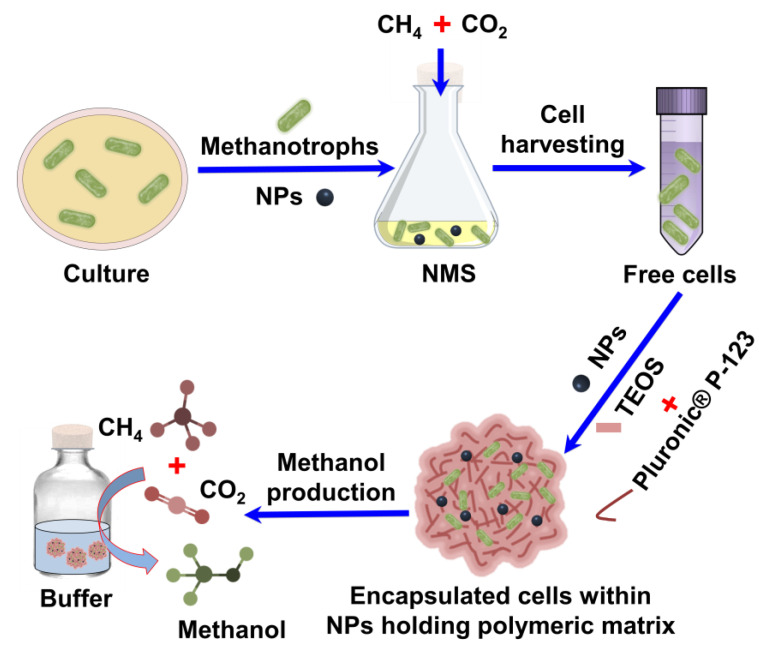
Schematic of methanotrophs grown in nitrate mineral salt (NMS) medium containing nanoparticles (NPs) using a simulated biogas [(methane (CH_4_) and carbon dioxide (CO_2_)) as feed to produce methanol by free and encapsulated cells within an NP-containing polymer matrix.

Polymers are well-known matrices with high biocompatibility for bioconversion applications in green synthesis [18]. Therefore, the encapsulation of methanotrophs within the pure polymeric matrix or embedded with NPs was evaluated to modulate methanol production from simulated biogas (Figure 2). The encapsulation of *M. bryophila* and *M. stellata* within a pure polymeric matrix produced slightly more methanol of 8.75 and 4.77 mmol/L, respectively, compared with the corresponding free-cell values of 8.18 and 4.63 mmol/L, respectively (Table 2). Previously, *M. trichosporium* and *M. sporium* strains within a polymeric matrix exhibited a maximum methanol concentration accumulation of up to 1.97 mmol/L [27]. In contrast, a slightly lower methanol production concentration of 1.94 mmol/L was observed in the PVA-immobilized *M. sporium* system [28]. The combination of various NPs (0.01 mg/mL) with polymers exhibited a much higher relative efficiency for methanol, up to 138, 124, and 141% for Cu, Fe_3_O_4,_ and CuFe_2_O_4_, respectively. The maximum methanol production was noted at 11.5 mmol/L (231 µmol) for *M. bryophila* and 5.79 mmol/L (116 µmol) for *M. stellata* using CuFe_2_O_4_ (0.01 mg/mL). Previously, a significantly lower efficiency for methanol production, between 40% and 68%, was reported for *M. trichosporium* immobilized on diethylaminoethyl cellulose, a mixed methanotroph culture on a ceramic ball, and for *Methylomonas* sp. immobilized on biomass-based coal and activated carbon [13,14,15]. The enhancement in methanol production or the stability of immobilized cells is primarily linked to MMO activity [11]. Higher relative MMO activities of 148 and 133% were observed for encapsulated cells over the corresponding *M. bryophila* (3.82 nmol/min/mg) and *M. stellata* (1.39 nmol/min/mg) free cells, respectively, linked within a polymeric matrix through cell stabilization or in the presence of Cu- and Fe-based NPs via a metal-dependent enhancement in the biocatalytic activity of methanotrophs. This is the first report of Cu or Fe NPs and a Pluronic^®^ P-123-based polymeric matrix for cell encapsulation.

**Figure 3:**
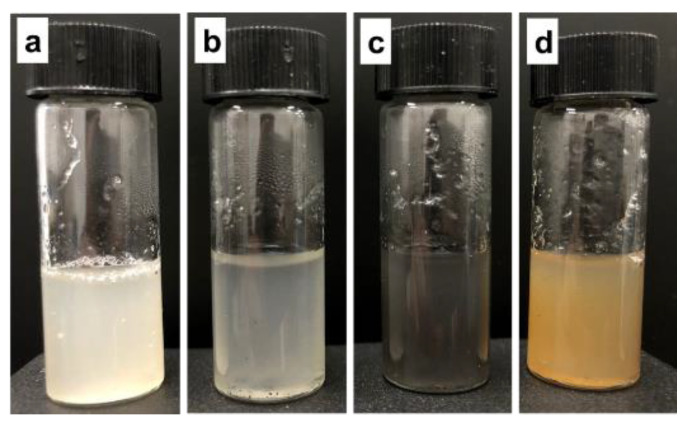
Photographs of encapsulated *M. bryophila* within an NP-containing polymer matrix: pure polymeric matrix (**a**), Cu–polymeric matrix (**b**), Fe_3_O_4_–polymeric matrix (**c**), and CuFe_2_O_4_–polymeric matrix (**d**).

**Figure 4:**
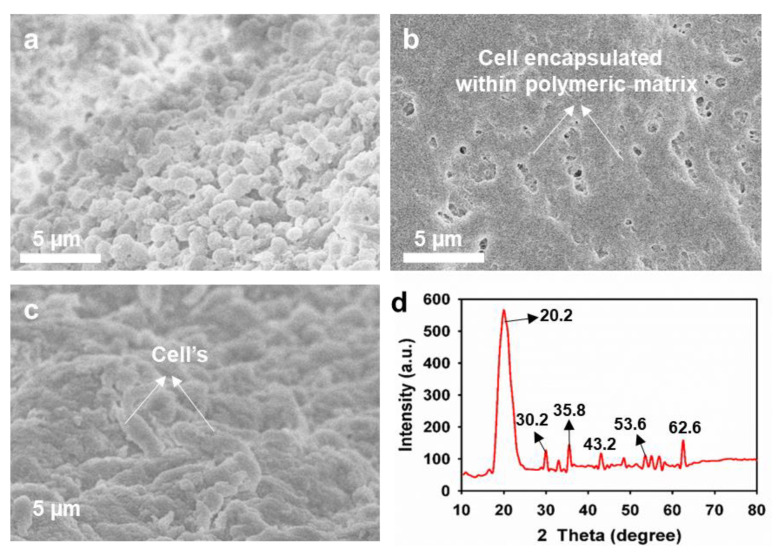
FE-SEM images of free and encapsulated cells for free *M. bryophila* (**a**), encapsulated cells within CuFe_2_O_4_–polymeric matrix (**b**), cross-sectional image of encapsulated cells (**c**), and XRD pattern of encapsulated cells within CuFe_2_O_4_–polymeric matrix (**d**).

Furthermore, optimization of the NP concentration was evaluated during encapsulation to determine its influence on methanol production (Table 3). An NP concentration of 0.025 mg/mL was found to be optimal during encapsulation for methanol production from simulated biogas. The maximum relative methanol production by encapsulated *M. bryophila* and *M. stellata* was up to 156% and 132%, with productions of 12.8 and 6.11 mmol/L and relative MMO activities of up to 167 and 143%, respectively, over methanol productions of 8.18 and 4.63 mmol/L and MMO activities of 3.82 and 1.39 nmol/min/mg, respectively, by their corresponding free cells. Previously, *M. stellata* and *M. trichosporium* OB3b produced much lower methanol concentration accumulation of up to 0.13 mmol/L from biogas [29]. An undefined mixed culture of methanotrophs grown on CH_4_ as an inoculum was reported to have a methanol concentration accumulation of 3.82 mmol/L [30]. In contrast, ammonia-metabolizing *Candidatus nitrosoglobus* exhibited a low methanol concentration accumulation of 1.5 mmol/L from CH_4_ [31]. The high methanol production with CuFe_2_O_4_ NPs compared with that with Cu or Fe_3_O_4_ NPs may be associated with the synchronized influence of these metals to preserve higher MMO activity (Table 3). The marginal decline in methanol production at a higher concentration (0.05 mg/mL) of these NPs resulted in a partial reduction in MMO activity or was associated with other physiological activity alterations in these methanotrophs [11]. The photographic images of encapsulated cells in a polymeric matrix containing NPs are presented in Figure 3. Furthermore, the encapsulation of *M. bryophila* within a polymeric matrix containing CuFe_2_O_4_ NPs was confirmed via FE-SEM measurements (Figure 4a–c). The XRD pattern exhibited peaks at 20.2 for silica and 30.2, 35.8, 43.2, 53.6, and 62.6 for CuFe_2_O_4_ (JCPDS 77–0010), which confirmed the formation of a silica-based CuFe_2_O_4_–polymeric matrix (Figure 4d) [32,33].

### 3.3. Influence of Physiological Parameters on Methanol Production by Encapsulated Methanotrophs

**Figure 5:**
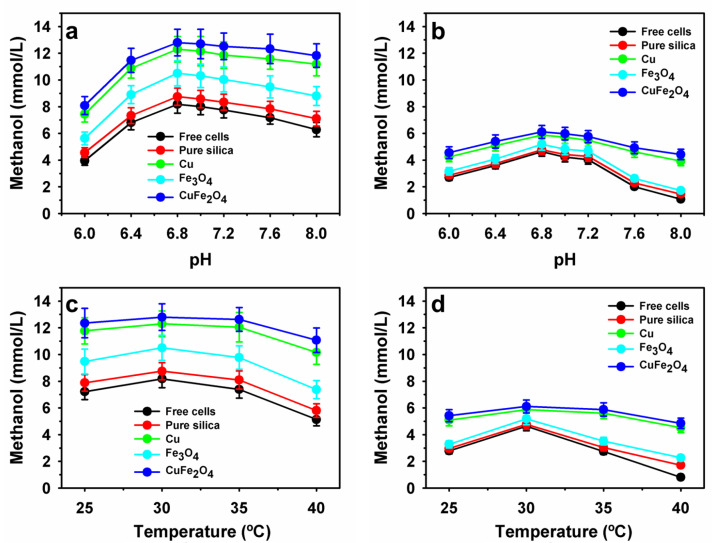
Effects of pH (**a**,**b**) and temperature (**c**,**d**) on methanol accumulation by methanotrophs encapsulated within an NP-based polymer matrix: *M. bryophila* (**a**,**c**) and *M. stellata* (**b**,**d**).

Immobilization is a suitable approach for improving the properties of whole-cell biocatalysts, particularly their physiological properties and recycling stability [11,15]. To evaluate the suitability of various NP-based encapsulated methanotroph systems, methanol production by free and encapsulated cells was compared at different pH values and temperatures (Figure 5). An optimum pH of 6.8 was noted for free and encapsulated *M. bryophila* and *M. stellata*. The cell residual methanol production was better maintained at lower and higher pH than their corresponding optimum values after encapsulation within the polymeric matrix (Figure 5a,b). The maximum residual methanol production improved for the *M. bryophila* CuFe_2_O_4_-based system to 8.09 and 11.8 mmol/L compared with the free cell values of 3.95 and 6.28 mmol/L at pH 6.0 and 8.0, respectively (Figure 5a). The various NPs-based encapsulated *M. bryophila* at these pH values retained higher residual methanol production of up to 30.8%. Under similar conditions, the CuFe_2_O_4_-based system of *M. stellata* exhibited a remarkable improvement in stability of as much as 312% (Figure 5b). Previously, a lower optimum pH of 3.0 was reported for *M. fumariolicum*, with methanol concentration accumulation of 4.10 mmol/L from CH_4_ [23]. Furthermore, an optimum temperature of 30 °C was observed for free and encapsulated *M. bryophila* and *M. stellata* (Figure 5c,d). Previously, a higher temperature optimum for methanol accumulation was reported at 37 °C and 55 °C for *Methylocaldum* sp. and *M. fumariolicum*, respectively [23,34]. The residual methanol production declined to 5.13 and 0.80 mmol/L at 40 °C in the case of free cells of *M. bryophila* and *M. stellata*, respectively. After encapsulation in a pure polymeric matrix, these methanotrophs exhibited residual methanol production of up to 5.81 mmol/L for *M. bryophila* and 1.71 mmol/L for *M. stellata*. At 40 °C, the various NP-based encapsulated systems of *M. bryophila* and *M. stellata* exhibited improvement of up to 1.4- and 4.6-fold compared with the corresponding free cells. Overall, the significant variations in the methanol production profiles after encapsulation may be associated with the various physiological properties of *M. bryophila* and *M. stellata*.

### 3.4. Production Profiles and Recycling of Encapsulated M. bryophila within Polymeric Matrix Containing CuFe_2_O_4_

**Figure 6:**
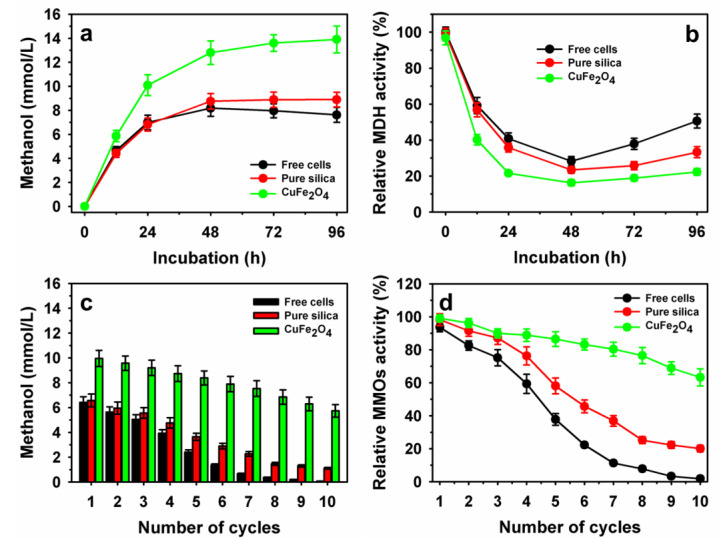
Methanol accumulation profile of free and encapsulated *M. bryophila* within a CuFe_2_O_4_–polymeric matrix (**a**), MDH activity (**b**), reusability (**c**), and MMO activity (**d**). The maximum biocatalytic activity was considered to be 100% for free or encapsulated cells.

The incubation period is highly influenced by the methanol accumulation by methanotrophs from CH_4_ due to the partial inhibition of MDH activity [8,11]. Methanol production by free cells increased to 8.18 mmol/L at 48 h and then declined to 7.62 mmol/L after 96 h of incubation (Figure 6a). This reduction in methanol accumulation was associated with a relatively higher gain in MDH activity to 50.6% at 96 h and over 28.3% after 48 h (Figure 6b). Previously, a remarkable decline of ~50% in accumulated methanol concentration was noted with a longer incubation of 72 h compared with the optimum incubation of 48 h (0.02 mmol/L) for *Methylosinus trichosporium* IMV 3011 using simulated biogas as the feed [26]. The pure polymeric matrix and CuFe_2_O_4_-based encapsulated *M. bryophila* exhibited maximum methanol accumulations of 8.87 and 13.9 mmol/L at 96 h of incubation. Greater methanol accumulation by these encapsulated cells was linked to the lower residual MDH activities of 33.3% and 22.3% for the polymeric matrix and CuFe_2_O_4_, respectively, compared with 50.6% for the free cells (Figure 6b). 

Reusability is an important criterion for assessing the success of immobilization procedures [11,27]. Therefore, the reusability of free and encapsulated cells was assessed 24 h after each cycle (Figure 6c,d). Free cells decreased the residual methanol production during subsequent cycles and nearly 99% of the initial methanol production (6.99 mmol/L) was lost after 10 cycles of reuse. The significant reduction in methanol production was linked to the preservation of almost 1.8% of the residual MMO activity. After encapsulation within pure polymeric matrix- and CuFe_2_O_4_-based systems, these cells retained high residual methanol production of 1.11 and 5.73 mmol/L, respectively, under similar recycling conditions. Here, the recycling stability after the encapsulation of *M. bryophila* improved 18.1-fold for the pure polymeric matrix and 62.5-fold for the CuFe_2_O_4_-based system. Previously, methanol production efficiency declined by >90% within three cycles of the repeated batch system for polymeric-matrix-immobilized *M. sporium* and *M. trichosporium* [27]. The preservation of greater residual methanol production by these encapsulated cells was accompanied by the retention of high residual MMO activity of up to 63.3% compared with 1.8% for free cells under repeated batch-mode conditions at 10 cycles. In contrast, cell-free silicone hydrogel-based systems of MMO biocatalysts may not be economically viable because of the requirement for expensive cofactors or the low stability of MMOs under non-natural conditions [35].

### 3.5. Influence of CH_4_ Vectors for Enhanced Methanol Production by Encapsulated M. bryophila

**Figure 7:**
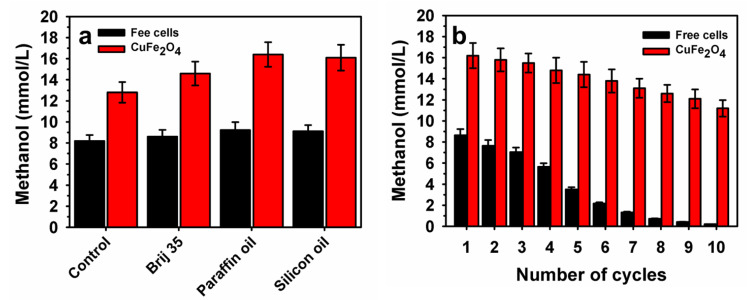
The influence of various CH_4_ vectors on methanol accumulation by *M. bryophila* within a CuFe_2_O_4_–polymeric matrix (**a**) and its reusability in the presence of paraffin oil (**b**).

The use of CH_4_ vectors can be beneficial for their conversion to methanol by methanotrophs by improving the solubility of CH_4_ [5,36]. To enhance methanol production by free and encapsulated *M. bryophila* within a polymeric matrix containing CuFe_2_O_4_, the effects of various CH_4_ vectors, including Brij 35, paraffin oil, and silicon oil, were assessed using simulated biogas as a feed for incubation for 48 h (Figure 7). These CH_4_ vectors positively influenced methanol production by both the free and encapsulated cells. In the case of free *M. bryophila*, there was an enhancement in methanol production to 8.60, 9.23, and 9.11 mmol/L in Brij 35, paraffin oil, and silicon oil, respectively, over the control value of 8.18 mmol/L (Figure 7a). The encapsulated cells exhibited a maximum methanol production of 16.4 mmol/L (329 µmol) in the presence of paraffin oil compared with the corresponding control value of 12.8 mmol/L (256 µmol). Previously, the maximum methanol concentration accumulation using engineered *M. trichosporium* OB3b (mxaF mutant) was reported to be 5.0 mmol/L using CH_4_ as the feed [12]. Overall, a higher methanol concentration accumulation in this study was noted than in the previous reports of various polymeric-matrices-based methanotrophs (Table S2). 

Furthermore, the reusability was evaluated in the presence of paraffin oil (5%, *v*/*v*) for repeated batch-mode methanol production for up to 10 cycles (Figure 7b). After 10 cycles, free and encapsulated cells retained a residual methanol production of 0.21 and 11.2 mmol/L, respectively. A nearly 20% improvement in residual methanol production was recorded compared with the value of 56.8% in the absence of a CH_4_ vector (Figure 6c) This finding suggested that reusability can be modulated for free and encapsulated cells in the presence of CH_4_ vectors to accumulate higher levels of methanol. The cells encapsulated within a polymeric matrix containing CuFe_2_O_4_ exhibited superior methanol production stability under repeated batch recycling by maintaining high biocatalytic activity compared with free cells. Overall, we developed a suitable strategy for renewable methanol production using NPs during inoculation preparation and encapsulation within polymeric matrix stages to enhance methanol production for the first time. Furthermore, the use of alternative feeds to simulated biogas, such as raw biogas derived from anaerobic digestion bioprocesses, is desirable for sustainable development using polymeric-matrix-and NP-based methanotroph systems [30,34]. 

## 4. Conclusions

The use of renewable energy sources derived from GHGs is a suitable GHG mitigation strategy. In this study, the use of Cu- and Fe-based NPs proved beneficial for the growth of methanotrophs as inocula in converting simulated biogas to methanol. Furthermore, polymeric-matrix- and NP-based encapsulation systems of methanotrophs exhibited significantly higher residual methanol production than the control. The cells encapsulated within a polymeric matrix containing CuFe_2_O_4_ exhibited superior methanol production stability under repeated batch recycling. The addition of CH_4_ vectors during bioconversion by encapsulated cells resulted in high methanol production potential and retained better recycling stability. These findings suggest that polymeric matrices and NPs are desirable for high methanol production from simulated biogas. In the future, employing bio-waste-derived raw biogas for growth and methanol production in the presence of NPs may become more viable for renewable energy generation by managing waste and GHGs.

## Figures and Tables

**Table 1:** Influence of feed and nanoparticles (NPs) on the methanotrophic growth to produce methanol from a simulated biogas.

NPs	Feed ^a^	Specific Growth Rate (h^−1^)		Methanol Production (mmol/L) ^b^	
		*M. bryophila*	*M. stellata*	*M. bryophila*	*M. stellata*
Control	CH_4_	0.013 ± 0.001	0.008 ± 0.001	4.90 ± 0.37	3.11 ± 0.26
	CH_4_ + CO_2_	0.015 ± 0.001	0.009 ± 0.001	5.45 ± 0.43	3.32 ± 0.22
Cu	CH_4_	0.016 ± 0.002	0.010 ± 0.001	6.60 ± 0.48	3.76 ± 0.25
	CH_4_ + CO_2_	0.019 ± 0.002	0.012 ± 0.001	7.25 ± 0.54	4.23 ± 0.33
Fe_3_O_4_	CH_4_	0.015 ± 0.001	0.009 ± 0.001	5.75 ± 0.49	3.42 ± 0.27
	CH_4_ + CO_2_	0.017 ± 0.002	0.010 ± 0.001	6.08 ± 0.52	3.54 ± 0.26
CuFe_2_O_4_	CH_4_	0.019 ± 0.002	0.012 ± 0.001	7.39 ± 0.56	4.26 ± 0.28
	CH_4_ + CO_2_	0.021 ± 0.002	0.015 ± 0.002	8.18 ± 0.67	4.63 ± 0.34

^a^ CH_4_ (30%) or simulated biogas (CH_4_ (30%) + CO_2_ (7.5%)) used as feed for up to 5 days of growth; ^b^ the feed used as simulated biogas containing CH_4_ (30%) and CO_2_ (15%) during methanol production for 48 h of incubation.

**Table 2:** Encapsulation of methanotrophs within NP-containing polymer matrix for methanol production.

Culture	Methanol Production (mmol/L)		MMO Activity (nmol/min/mg)	
	*M. bryophila*	*M. stellata*	*M. bryophila*	*M. stellata*
Free cells	8.18 ± 0.67	4.63 ± 0.34	3.82 ± 0.26	1.39 ± 0.09
Pure polymeric matrix	8.75 ± 0.64	4.77 ± 0.37	4.43 ± 0.33	1.51 ± 0.11
Cu–polymeric matrix	11.3 ± 0.87	5.60 ± 0.42	5.54 ± 0.37	1.71 ± 0.12
Fe_3_O_4_–polymeric matrix	10.1 ± 0.76	5.05 ± 0.38	5.01 ± 0.38	1.58 ± 0.11
CuFe_2_O_4_–polymeric matrix	11.5 ± 0.85	5.79 ± 0.44	5.65 ± 0.42	1.85 ± 0.13

**Table 3:** Influence of NP concentration during encapsulation of methanotrophs within polymeric matrix to produce methanol from simulated biogas.

NPs	NP Conc. (mg/mL)	Methanol Production (mmol/L)		MMO Activity (nmol/min/mg)	
		*M. bryophila*	*M. stellata*	*M. bryophila*	*M. stellata*
Control (free cells)	0	8.18 ± 0.67	4.63 ± 0.34	3.82 ± 0.26	1.39 ± 0.09
Cu	0.010	11.3 ± 0.87	5.60 ± 0.42	5.54 ± 0.37	1.71 ± 0.12
	0.025	12.3 ± 0.96	5.88 ± 0.45	6.15 ± 0.48	1.90 ± 0.15
	0.050	11.9 ± 0.88	5.69 ± 0.39	5.81 ± 0.46	1.74 ± 0.14
Fe_3_O_4_	0.010	10.1 ± 0.76	5.05 ± 0.38	5.01 ± 0.38	1.58 ± 0.11
	0.025	10.5 ± 0.83	5.19 ± 0.44	5.08 ± 0.44	1.63 ± 0.13
	0.050	10.4 ± 0.79	5.14 ± 0.41	5.04 ± 0.39	1.58 ± 0.12
CuFe_2_O_4_	0.010	11.5 ± 0.85	5.79 ± 0.45	5.65 ± 0.42	1.85 ± 0.13
	0.025	12.8 ± 0.99	6.11 ± 0.49	6.38 ± 0.48	1.99 ± 0.15
	0.050	12.4 ± 0.91	5.88 ± 0.48	6.11 ± 0.45	1.89 ± 0.15

## Supplementary Materials

The following supporting information can be downloaded at: https://www.mdpi.com/article/10.3390/polym15183667/s1, Table S1: The utilization of methane during the growth of methanotrophs in NMS media in the presence of nanoparticles (NPs); Table S2: A comparison of methanol production by encapsulated methanotrophs within a polymeric matrix; Figure S1: The methanol production profiles of methanotrophs cultured in the presence of various nanoparticles from simulated biogas as feed: *M. bryophila* grown in CH4 (a) and simulated biogas (b), and M. stellata grown in CH4 (c) and simulated biogas (d).
