# Supplementary material for: Encapsulation of Methanotrophs within a Polymeric Matrix Containing Copper- and Iron-Based Nanoparticles to Enhance Methanol Production from a Simulated Biogas
Source: Polymers (Basel). 2023 Sep 6;15(18):3667. doi: 10.3390/polym15183667 (PMC10537138; doi:10.3390/polym15183667)
Supplement: Supplementary file 1 [file polymers-15-03667-s001.zip › polymers-2558489-supplementary.pdf]

polymers

Supporting Materials

Encapsulation of methanotrophs within a polymeric matrix containing copper- and iron-based nanoparticles to enhance methanol production from a simulated biogas

Sanjay K. S. Patel [1], Rahul K. Gupta [1], In-Won Kim [1,*], and Jung-Kul Lee [1,*]

[1] Department of Chemical Engineering, Konkuk University, 120 Neungdong-ro, Gwangjin-gu, Seoul 05029, Republic of Korea

* *Correspondence: jkrhee@konkuk.ac.kr (J.-K.L.); Tel.: +82-2-450-3505*

Table S1. The utilization of methane during the growth of methanotrophs in NMS media in the presence of nanoparticles (NPs).

NPs	Feed [a]	Substrate utilization (%)	
		M. bryophila	*M. stellata*
Control	CH_4	22.4 ± 1.3	17.9 ± 1.5
(Without NPs)	$CH_4 + CO_2$	23.7 ± 1.6	18.1 ± 1.4
Cu	CH_4	31.8 ± 2.3	19.6 ± 1.6
	$CH_4 + CO_2$	32.3 ± 2.5	20.2 ± 1.5
Fe_3O_4	CH_4	28.6 ± 2.2	18.2 ± 1.3
	$CH_4 + CO_2$	30.7 ± 2.6	18.5 ± 1.4
$CuFe_2O_4$	CH_4	33.1 ± 2.7	19.8 ± 1.6
	$CH_4 + CO_2$	35.4 ± 2.9	23.3 ± 1.8

[a] CH_4 (30%) or simulated biogas [CH_4 (30%) + CO_2 (7.5%) was used as feed for up to 5 days of growth.

Table S2. A comparison of methanol production by encapsulated methanotrophs within the polymeric matrix.

Support	Culture	Accumulated methanol concentration (mmol/L)	Reference
Polymer matrix	*Methylosinus sporium* B-2119	1.68	[27]
	M. sporium B-2120	1.43	
	M. sporium B-2121	2.34	
	M. sporium B-2122	1.37	
	M. sporium B-2123	1.84	
Polyvinyl alcohol	*M. sporium* B-2121	1.94	[28]
Sodium-alginate	*Methylosinus trichosporium* OB3b	3.70	[11]
$CuFe_2O_4$-polymeric matrix	*M. bryophila*	16.4	This study

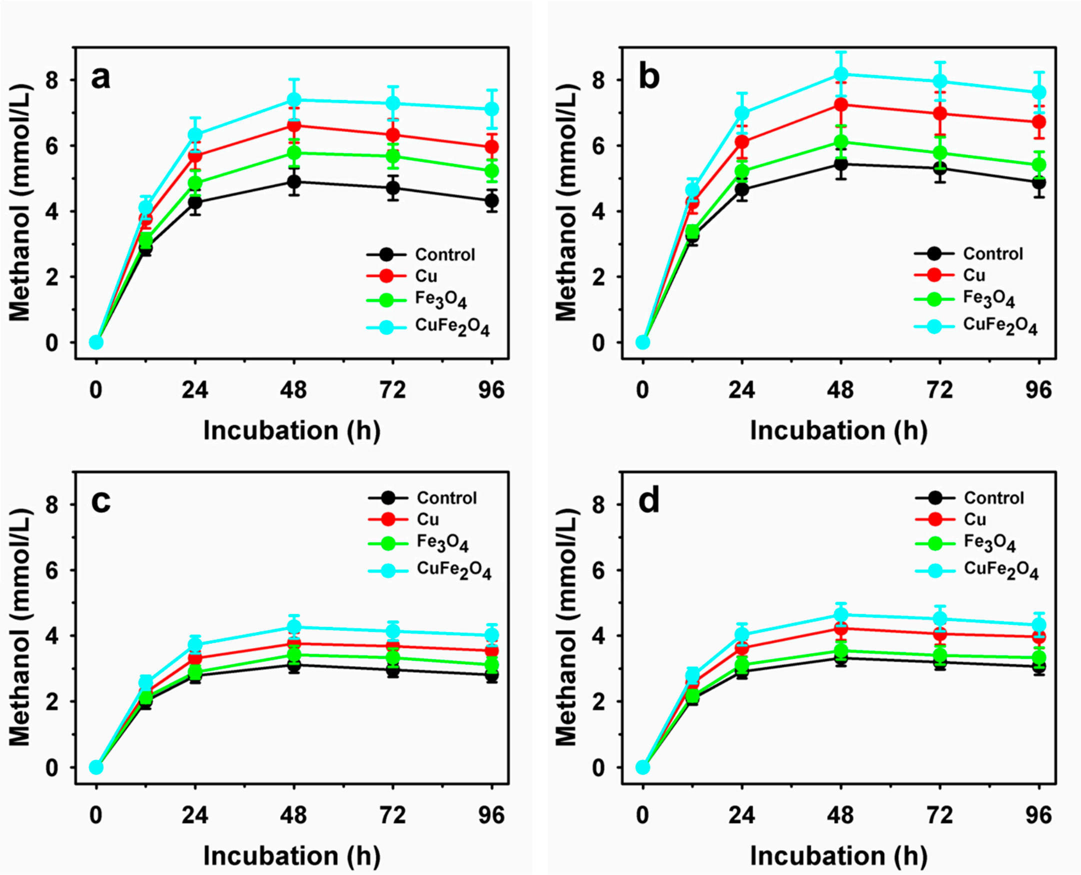

Figure S1. The methanol production profiles of methanotrophs cultured in the presence of various nanoparticles from simulated biogas as feed: *M. bryophila* grown in CH_4 (a) and simulated biogas (b), and *M. stellata* grown in CH_4 (c) and simulated biogas (d).
